# When the Abdomen Cannot Be Closed: From Open Abdomen and Enteroatmospheric Fistula to Definitive Abdominal Wall Reconstruction

**DOI:** 10.7759/cureus.117927

**Published:** 2026-10-14

**Authors:** Daniel Gravino, Matthew Debattista, Kyle Debono

**Affiliations:** 1 Surgery, Mater Dei Hospital, Msida, MLT

**Keywords:** abdominal wall reconstruction, component separation, enteroatmospheric fistula, enterocutaneous fistula, mesh, negative-pressure wound therapy, open abdomen

## Abstract

The open abdomen can be a life-saving intervention in selected patients with abdominal compartment syndrome, major trauma, severe intra-abdominal sepsis, or a continuing need for re-exploration. It also initiates a complex reconstructive pathway in which fascial retraction, visceral fixation, intestinal failure, and enteroatmospheric fistula may compromise eventual recovery. This narrative review integrates initial open-abdomen management, fistula control, intestinal rehabilitation, and definitive abdominal wall reconstruction, with particular attention to the applicability and limitations of the evidence at each transition. Early priorities are protection of exposed viscera and pursuit of safe primary fascial closure once resuscitation, source control, and bowel viability permit. Negative-pressure wound therapy with a fascial traction system may improve closure in selected patients, but the recommendation is conditional and rests largely on low-certainty observational evidence. When enteroatmospheric fistula develops, treatment centers on sepsis control, separation of enteric effluent from the wound, replacement of measured losses, and individualized nutritional support. Definitive intestinal surgery should be timed to clinical and biological readiness rather than a universal minimum interval. Simultaneous fistula takedown and abdominal wall reconstruction is feasible in selected patients but carries substantial wound morbidity and recurrence; staged treatment remains appropriate when contamination, tissue quality, or physiological reserve is unfavorable. Reconstruction prioritizes restoration of fascial continuity, protected mesh reinforcement when appropriate, and reliable soft-tissue coverage. Randomized evidence, including long-term follow-up, favors permanent synthetic over biologic mesh in selected clean-contaminated and contaminated ventral hernia repairs, but does not establish safety in an actively septic open abdomen with uncontrolled enteric leakage. The principal contribution of this review is a connected, evidence-calibrated pathway that distinguishes direct open-abdomen and fistula evidence from extrapolation and emphasizes intestinal autonomy, abdominal wall function, and patient experience alongside anatomical closure.

## Introduction and background

An abdomen is left open when definitive closure would compromise physiology or prevent necessary further treatment. Relevant indications include abdominal compartment syndrome, damage-control surgery, selected cases of severe abdominal sepsis, uncertain bowel viability, and planned re-exploration. The open abdomen (OA) provides access and relieves pressure, but continued exposure risks fascial retraction, visceral adhesions, fluid and protein losses, and fistulation. Guidance therefore treats OA as a temporary strategy and prioritizes closure once the indication has resolved. The associated morbidity, repeated procedures, and prolonged hospitalization impose a substantial burden on patients and their recovery [1,2].

The amended Björck classification distinguishes contamination, developing fixation, a frozen abdomen, and established enteroatmospheric fistula (EAF). It describes anatomical complexity rather than determining treatment by itself [3]. An EAF is an opening from bowel directly into an exposed abdominal wound, without the mature soft-tissue tract typical of an enterocutaneous fistula (ECF). Spontaneous closure is particularly unlikely when mucosa protrudes or bowel is fixed within the wound; occasional closure reported in selected series should not be generalized to all EAFs [4].

Existing guidelines and comprehensive reviews already address major elements of OA care. The remaining clinical problem is how to connect decisions across phases that are usually studied separately: temporary closure, EAF isolation, intestinal rehabilitation, bowel reconstruction, and restoration of a functional abdominal wall. Moreover, evidence from elective hernia repair or tract-bearing ECF is not automatically applicable to active EAF. This review adds an explicit appraisal of those transitions, identifying how early tissue preservation affects later reconstructive options and where treatment recommendations require extrapolation [2,5].

## Review

Literature selection, scope, and evidence interpretation

This targeted narrative review was revised using web-assisted retrieval of PubMed/MEDLINE-indexed records, PubMed Central articles, original journal pages, and professional society guidance, supplemented by reference tracing. The eligible publication period had no lower date limit, and retrieval was completed on 6 October 2026. Contemporary sources from 2016 onward were prioritized for clinical recommendations; earlier original descriptions were retained selectively for the provenance of reconstructive techniques. The principal Boolean combinations used for targeted retrieval are provided in Table 1.

**Table 1:** Search framework for the revised narrative review OA: open abdomen; DOI: digital object identifier. Boolean combinations were used for targeted web-assisted retrieval of indexed records; this is not a claim of exhaustive database coverage.

Search component	Retrieval approach
Publication period	No lower publication-date limit; retrieval completed on 6 October 2026
OA and temporary closure	("open abdomen" OR laparostomy) AND ("temporary abdominal closure" OR "fascial traction" OR "visceral protective layer")
Fistula control and nutrition	("enteroatmospheric fistula" OR "enterocutaneous fistula") AND (isolation OR nutrition OR "negative pressure")
Timing and intestinal reconstruction	("enteric fistula" OR "enterocutaneous fistula") AND ("definitive surgery" OR timing OR recurrence OR "abdominal wall reconstruction")
Definitive wall reconstruction	("contaminated ventral hernia" OR "abdominal wall reconstruction") AND (randomized OR mesh OR "transversus abdominis release" OR flap)
Supplementary retrieval	Searches by article title, author, and DOI; original publisher and society pages; reference tracing; targeted searches for newer follow-up reports
Selection and verification	Single, non-duplicate purposive selection; full texts assessed when accessible; numerical findings and bibliographic metadata checked against original or indexed records

This approach did not constitute an exhaustive direct search of every bibliographic database.

Titles and abstracts were assessed for relevance to OA, EAF/ECF, intestinal failure, or complex reconstruction. Potentially relevant sources were examined in full when accessible; otherwise, interpretation was restricted to the indexed abstract and bibliographic record. Selection followed a single, non-duplicate screening stream; independent dual screening was not undertaken. Sources were chosen purposively for clinical relevance, methodological strength, population applicability, and additional information beyond overlapping reports. Follow-up reports from the same trial were linked rather than treated as independent patient cohorts. Eligibility criteria are summarized in Table 2.

**Table 2:** Eligibility and applicability criteria OA: open abdomen; EAF: enteroatmospheric fistula; ECF: enterocutaneous fistula.

Category	Criteria
Population	Adult OA, EAF/ECF, intestinal failure, or complex abdominal wall reconstruction; mixed cohorts considered only when relevant adult evidence could be interpreted
Eligible designs	Guidelines, consensus statements, randomized trials, systematic reviews, registries, comparative cohorts, and relevant technical or reconstructive series
Indirect evidence	Elective hernia or broader ECF evidence included only with explicit explanation of its relevance and limits for OA/EAF
Historical exceptions	Original descriptions of component separation and transversus abdominis release retained to establish technical provenance
Exclusions	Pediatric-only and animal studies; abstract-only conference reports used to support recommendations; isolated case reports when adequately represented by broader evidence; uncomplicated hernia studies without relevant reconstructive information

The final bibliography contained 39 sources; this was a reference count, not a systematic-review study count. An exhaustive retrieval denominator, duplicate-removal count, and screening flow were not compiled. The review was not protocol-registered and does not claim Preferred Reporting Items for Systematic Reviews and Meta-Analyses (PRISMA) compliance. Formal study-level risk-of-bias scoring or Grading of Recommendations Assessment, Development, and Evaluation (GRADE) assessment was not performed.

Instead, key statements identify their evidence basis as randomized comparative evidence, observational evidence or its synthesis, guideline/consensus, or technical evidence. These descriptors are not interchangeable with certainty ratings: a meta-analysis of observational series remains vulnerable to confounding, and a strong guideline recommendation may rely on limited direct evidence. Observational studies vary in patient selection, fistula anatomy, contamination, operative technique, outcome definitions, and follow-up, limiting comparability and the generalizability of their findings. Numerical results were reported from the original studies without new pooling or cross-study statistical comparison. In this narrative synthesis, published pooled estimates were used descriptively to summarize the studied populations, rather than as validated predictions for an individual patient or a different clinical setting. Their confidence intervals express uncertainty around the pooled estimate and should not be interpreted as individual prediction intervals.

First operation is already reconstructive

Early care should preserve a route to fascia-to-fascia closure. Contemporary reviews describe closure within approximately five to seven days as a desirable opportunity when physiology permits, rather than a deadline that overrides source control or pressure concerns [5]. Temporary abdominal closure should limit injury, evacuate fluid, and prevent avoidable fascial retraction.

The Eastern Association for the Surgery of Trauma (EAST) conditionally recommends a fascial traction system in hemodynamically stable trauma and emergency general surgery patients after the intra-abdominal pathology has been addressed. Its evidence assessment was very low certainty. Traction should therefore be considered in suitable patients by teams familiar with the method, rather than prescribed universally [6]. A 2024 single-arm meta-analysis of vacuum-assisted closure with mesh-mediated traction included seven studies and 535 patients. It supports feasibility but cannot establish comparative superiority, and heterogeneity limits translation of pooled outcomes to individual patients [7].

A visceral protective layer separates the bowel from potentially injurious dressing or traction materials. Observational findings from the European Hernia Society Open Abdomen Registry associate its use with fewer EAFs. This is direct OA registry evidence, although its design cannot establish that the protective layer alone prevents fistulation [8]. Visceral protection should be maintained during each dressing change. Foam, drains, or traction mesh should not apply focal pressure directly to unprotected bowel. Re-exploration is generally undertaken within 24-48 hours when indicated, with reassessment of bowel viability, source control, and the opportunity for closure [1].

Fluid treatment must sustain perfusion while avoiding unnecessary accumulation that worsens visceral edema. Repeated laparotomy, bowel injury, prolonged exposure, and persistent contamination complicate subsequent closure. As a practical planning inference, the operative record should describe remaining fascia, incisions, stomas, bowel continuity, and areas of tissue injury so that later teams can preserve useful planes and vascular supply [2].

When an EAF occurs

Initial treatment aims to stabilize the patient and make wound contamination controllable. Effluent output, fistula location, mucosal protrusion, surrounding bowel exposure, and wound geometry should be documented. Uncontrolled sepsis requires imaging and drainage or surgery according to the source; a controlled fistula does not justify ignoring an undrained intra-abdominal collection [4].

The reconstructive task is to separate enteric drainage from the remaining wound. Techniques can be grouped into stoma-based diversion, barrier or adapter systems, and fistula-isolating negative-pressure dressings. An appliance that works for protruding mucosa may fail for a flat fistula in a deep wound. The available literature is largely technical or observational, and no isolation device has established universal superiority [6]. Negative pressure can support the surrounding wound when bowel is protected and the fistula is separately drained; it should not be interpreted as a validated method for closing every EAF. Local suturing of an inflamed, fixed opening is often unsuccessful and may cause further injury [4].

Isolation also creates practical endpoints that should be monitored: dressing durability, skin injury, ability to measure output, freedom from recurrent pooling, and tolerance of mobilization and feeding. These are proposed process measures rather than a validated outcome score. The best configuration is the one that reliably controls the particular wound while preserving tissue for later reconstruction.

Intestinal rehabilitation and physiological recovery

Fluid, electrolyte, and nutritional support must respond to measured losses and residual intestinal function. Proximal high-output fistulas can cause substantial sodium, magnesium, water, and nutrient depletion. Monitoring should include renal function, electrolyte trends, output, intake, weight trajectory, and evidence of dehydration or fluid overload; replacement cannot be reduced to a generic maintenance prescription [9,10].

American Society for Parenteral and Enteral Nutrition-Latin American Federation of Nutritional Therapy, Clinical Nutrition and Metabolism (ASPEN-FELANPE) guidance supports individualized enteral, parenteral, or combined nutrition [11]. A contemporary nutrition review emphasizes that high-output ECF, commonly defined as more than 500 mL/day, may require parenteral nutrition, while oral or enteral intake may become feasible when drainage is controlled and requirements can be met. Output alone is insufficient to select a route: location, continuity, distal obstruction, absorptive length, and the response to feeding also matter [12].

Distal feeding or reinfusion strategies may be considered when a patent downstream segment is accessible and the specialist team can establish a safe protocol. Evidence and expertise from intestinal failure services are relevant, but an ECF feeding pathway should not be transferred uncritically to exposed, unstable bowel. Long-term parenteral support requires attention to venous access, infection, liver and metabolic complications, and the patient's ability to manage treatment outside hospital [13].

Albumin and prealbumin should not be treated as stand-alone measures of nutritional adequacy or mandatory thresholds for surgery. They respond to inflammation and do not reliably quantify body protein or muscle mass [14]. Recovery is better judged by the combination of nutritional delivery, clinical examination, functional improvement, correction of deficiencies, inflammatory course, and manageable fluid losses. Rehabilitation should include mobilization, respiratory conditioning, and support for the psychological burden of prolonged illness.

Readiness for definitive intestinal surgery

Elective fistula reconstruction should follow control of infection and recovery from severe catabolism. In a systematic review of 15 studies of reconstructive intestinal failure surgery, longer intervals to surgery were associated with lower recurrence, but the optimal interval could not be defined. The association was derived from heterogeneous observational cohorts and does not establish a universal minimum delay [15].

For complex EAF, World Society of Emergency Surgery (WSES) guidance describes postponement until recovery and wound healing, often for at least six months. This is consensus-informed guidance for a difficult population, not proof that six months is a biological threshold [1]. Timing should therefore be individualized: persistent collections, active inflammation, an unsafe access plane, unstable losses, or poor functional reserve may justify further delay. Conversely, obstruction, ischemia, or failure of source control may require earlier intervention for the immediate problem, without attempting complete elective reconstruction.

The term "wound maturation" means a stable, controlled wound with improved tissue quality and a safer dissection plane. It does not imply that an EAF must stop draining. In a skin-grafted abdomen, clinical mobility of the graft relative to underlying bowel may help assess entry, but no single bedside finding guarantees safe adhesiolysis [2].

Cross-sectional imaging should answer specific questions about bowel continuity, fistula-bearing segments, collections, existing mesh, stomas, abdominal wall tissue, and defect geometry. Nutritional and physical preparation should be active rather than passive waiting. Contemporary abdominal wall prehabilitation literature supports assessment of smoking, metabolic control, frailty, and exercise capacity; its predominantly elective population limits certainty about the optimal program for intestinal failure [16].

The phase-specific pathway in Table 3 distinguishes the evidence supporting these transitions.

**Table 3:** Phase-specific priorities and the evidence supporting them This synthesis does not assign new formal Grading of Recommendations Assessment, Development, and Evaluation (GRADE) ratings. OA: open abdomen; EAF: enteroatmospheric fistula; NPWT: negative-pressure wound therapy; EAST: Eastern Association for the Surgery of Trauma.

Phase	Main objective	Evidence-calibrated actions	Basis and applicability
Index operation and temporary closure	Preserve survival and a route to later closure	Protect viscera; preserve fascia; consider NPWT with a traction system once physiology and source control permit	Guideline/consensus; EAST traction recommendation is conditional with very low-certainty evidence [6,8]
Reassessment and re-exploration	Resolve the OA indication and close safely	Reassess viability, infection, and pressure; close promptly when safe; avoid using a day-count target alone	Guidance and observational synthesis; early closure is conditional on recovery [5]
Established EAF	Control sepsis and drainage	Isolate effluent; protect the wound; quantify losses; individualize nutritional support	Mainly technical/observational fistula evidence and nutrition guidance [9,11,12]
Rehabilitation and planning	Establish clinical and anatomical readiness	Resolve infection, stabilize losses, improve function, and map anatomy; individualize the interval to surgery	Observational timing evidence and expert practice; no universal minimum interval established [15,16]
Bowel and wall reconstruction	Restore continuity and durable coverage	Select combined or staged treatment; pursue feasible fascial restoration and appropriate reinforcement; use flaps when tissue is deficient	Combined-repair and flap evidence is predominantly observational; randomized mesh evidence applies to selected controlled fields [17-19]
Long-term follow-up	Assess durability and patient recovery	Record fistula, hernia, support requirements, reoperation, symptoms, and function separately	Longitudinal observational evidence; early anatomical closure alone is insufficient [20]

Simultaneous versus staged reconstruction

Combined intestinal and abdominal wall reconstruction can restore continuity and cover the viscera within one operation, but it concentrates bowel and wound risks into the same procedure. Selection should consider physiological reserve, the expected adhesiolysis burden, control of contamination, remaining bowel length, availability of fascia, and reliability of soft-tissue coverage.

In the Abdominal Core Health Quality Collaborative (ACHQC) registry, 170 patients undergoing concomitant elective ECF and hernia repair had 30-day surgical-site infection of 18% and surgical-site occurrences requiring procedural intervention of 21%. These are short-term registry outcomes from selected elective ECF-hernia reconstruction, with limited applicability to active EAF or acute OA care [20,21]. A 2026 single-arm meta-analysis of nine studies and 697 patients undergoing simultaneous ECF treatment and wall repair reported surgical-site infection of 41% (95% confidence interval or CI 28%-56%), hernia recurrence of 27% (95% CI 19%-37%), and fistula recurrence of 9% (95% CI 5%-16%). Follow-up and techniques varied substantially, and high heterogeneity restricts individual risk prediction [16].

These predominantly observational reports show feasibility with appreciable morbidity; neither directly establishes superiority over staged treatment. Patients selected for simultaneous reconstruction may differ from those requiring staged treatment in physiological reserve, contamination control, fistula complexity, and available tissue. Such selection differences create confounding by indication and limit direct comparison of reported outcomes. A staged pathway may be appropriate when bowel repair is necessary but definitive prosthetic reinforcement or soft-tissue reconstruction is unsafe. It can separate resolution of enteric contamination from subsequent wall reconstruction, at the cost of another operation and continued hernia-related disability. Such trade-offs remain a multidisciplinary judgment because comparative evidence is insufficient [16].

Definitive bowel surgery and restoration of fascial continuity

The bowel operation should be planned from the anatomy and the amount of functional intestine that can be preserved. Adequate exposure and careful adhesiolysis reduce the risk of overlooked enterotomies. Fistula-bearing diseased bowel is generally resected when feasible; local repair remains a selected option rather than a default. A 2026 specialist-center cohort of 93 ECF repairs reported 24% recurrence and 5% mortality. Primary repair was independently associated with recurrence (odds ratio (OR) 4.55; 95% CI 1.10-18.85), but residual confounding by case complexity prevents interpreting this as a randomized comparison [22].

Decisions about restoring intestinal continuity or creating a diverting stoma should consider bowel viability, distal patency, physiological stability, and remaining bowel length. Before permanent mesh reinforcement is considered, the team should reassess residual contamination and the suitability of the bowel reconstruction; completion of fistula takedown should not itself be regarded as establishing a clean operative field [2,20].

The abdominal wall objective is restoration of midline continuity with acceptable tension. Reinforcement of a closed fascia and prosthetic bridging are different operations: a bridge spans a defect without re-approximating its fascial edges. European Hernia Society guidance favors defect closure and retromuscular mesh positioning in applicable incisional hernias. However, that guideline primarily addresses hernias expected to close without advanced release, so extension to complex EAF reconstruction is an anatomical principle rather than direct evidence for every hostile abdomen [23].

Choosing a myofascial release

Anterior component separation advances abdominal wall tissue after release of the external oblique aponeurosis; its original description remains relevant for technical provenance [24]. Extensive classic undermining can jeopardize skin perfusion. Contemporary perforator-preserving approaches aim to maintain vascular supply and should be distinguished from older open techniques when comparing morbidity [25].

Posterior component separation with transversus abdominis release (TAR) expands the retromuscular space and facilitates medial advancement and broad reinforcement without large anterior skin flaps [26]. A 2025 systematic review suggests lower wound morbidity with posterior/TAR approaches compared with anterior separation, but variation in anterior techniques and case selection limits a universal hierarchy. The 2023 perforator-preserving cohort found no statistically significant difference in overall wound complications between its anterior and TAR groups, illustrating why a technique label alone is inadequate for comparison [25,27].

A 2026 meta-analysis of 10 observational studies involving 1,085 patients associated robotic TAR with fewer surgical-site occurrences (OR 0.48; 95% CI 0.28-0.80) and shorter stay (mean difference -4.65 days; 95% CI -5.65 to -3.66) than open TAR. Operating time was longer, and recurrence did not differ significantly (OR 0.89; 95% CI 0.30-2.63). These data concern selected ventral hernia repairs and cannot establish benefit for active EAF with extensive adhesiolysis [28]. The required release should be chosen from the viable anatomy, existing surgical planes, and achievable closure; robotic access is a selected delayed option rather than an obligatory endpoint.

Mesh selection in controlled contamination

The highest-quality comparative mesh evidence relates to selected clean-contaminated and contaminated ventral hernias. In a multicenter randomized trial of 253 patients receiving retromuscular reinforcement at fascial closure, two-year recurrence was 5.6% with synthetic mesh and 20.5% with biologic mesh. Synthetic mesh reduced recurrence risk (hazard ratio or HR 0.31; 95% CI 0.23-0.42), with no significant difference in surgical-site occurrences requiring intervention and substantially lower cost [18].

Long-term follow-up is now available. The linked 2026 report obtained follow-up in 203 patients (80.2%), with a median duration of 5.4 years. Reported long-term midline recurrence was 11.8% with synthetic mesh and 23.6% with biologic mesh (HR 0.46; 95% CI 0.25-0.86). No new mesh infections or excisions occurred in either group beyond two years. Attrition and the selected operative setting remain relevant limitations [29]. These are two reports from the same randomized cohort, not independent trials.

The findings favor permanent synthetic reinforcement in the studied setting; they do not test its safety in a persistently septic OA, uncontrolled enteric leak, unprotected bowel contact, or an unclosed fascial gap. Field control, a suitable plane, anastomotic reliability, and coverage must therefore be assessed separately. A propensity-matched Abdominal Core Health Quality Collaborative (ACHQC) registry analysis informs permanent versus absorbable mesh selection in elective clean-contaminated (class II) and contaminated (class III) ventral hernia repair. This is hernia reconstruction evidence rather than direct evidence for an actively septic OA or uncontrolled EAF, and matching cannot remove all treatment-selection bias [30].

Biosynthetic mesh may be considered when a resorbable scaffold is desirable, without assuming proven superiority. A 2023 systematic review included 21 reports and 1,619 patients, but mesh types, contamination, and follow-up varied; observed recurrence increased with longer follow-up [31]. The prospective Complex Open Bioabsorbable Reconstruction of the Abdominal Wall (COBRA) study supplies complementary cohort evidence for biosynthetic absorbable mesh in selected contaminated reconstructions; its design does not resolve comparative effectiveness [32]. Table 4 summarizes the practical implications of these distinct evidence bases.

**Table 4:** Selection of definitive abdominal wall strategies and the applicability of their supporting evidence TAR: transversus abdominis release; EAF: enteroatmospheric fistula.

Strategy	Suitable setting	Evidence and principal limitations
Closed fascia with retromuscular permanent synthetic reinforcement	Achievable fascial closure, controlled contamination, suitable tissue planes, reliable coverage	Lower recurrence than biologic mesh in the cited randomized trial and its linked long-term report; not established for uncontrolled EAF or unprotected bowel [18,29]
Posterior component separation/TAR	Medial advancement and a broad retromuscular plane are required and can be safely developed	Technical and comparative evidence; prior surgery may eliminate useful planes; release remains anatomy-dependent [26,27]
Robotic TAR	Selected elective or delayed reconstruction after contamination has resolved	Observational synthesis associates it with less wound morbidity and shorter stay, but longer operating time; no established advantage in active EAF [28]
Perforator-preserving anterior component separation	Anterior advancement is appropriate and skin perfusion can be maintained	Cohort evidence; results should not be equated with classic wide-undermining anterior separation [25]
Biosynthetic absorbable reinforcement	Selected reconstruction where a resorbable scaffold is chosen	Observational synthesis and prospective cohort evidence; comparative durability and material-specific effects remain uncertain [31,32]
Biologic reinforcement	An individualized indication after considering field, plane, alternatives, and expected durability	No recurrence advantage over synthetic mesh in the cited randomized setting; not a solution to ongoing leakage [18,29]
Vascularized flap reconstruction	Deficient or unreliable soft tissue, dead space, or composite/full-thickness tissue loss	Technical series and heterogeneous reviews; fascial or structural requirements must be addressed separately from skin coverage [19,33,34]
Planned ventral hernia or staged wall reconstruction	Definitive closure would be unsafe or an adequate repair cannot yet be constructed	Guideline/consensus and observational experience; accepts prolonged disability and later reconstruction [2]

Soft-tissue reconstruction and the plastic surgeon's role

Soft-tissue coverage and load-bearing reconstruction solve related but different problems. Fascia and reinforcement address structural continuity; local or transferred tissue supplies durable coverage, fills dead space, and replaces scarred or missing tissue. Local advancement may suffice when viable skin remains. Composite defects may require regional or free tissue, selected by location, pedicle reach, recipient vessels, previous incisions, donor morbidity, and whether fascia or muscle is required [33].

A 2025 systematic review of 104 free flaps reported no total flap losses, but its small, selected literature and predominance of oncological indications limit estimates of safety for fistulating abdominal wounds [34]. A broader 2026 review identified 172 studies and 4,421 patients; pedicled techniques predominated, and approaches varied markedly by defect composition. Its findings support defect-specific planning rather than establishing one superior flap [19].

Direct fistula-related evidence is more limited. A pilot study examined fasciocutaneous anterolateral thigh flaps combined with structural repair following ECF resection, demonstrating a relevant reconstructive strategy without establishing superiority over other coverage methods [35]. Perforator preservation and prospective coordination of stomas, incisions, and donor sites are therefore central planning tasks. A flap containing fascia can contribute structural tissue; a skin flap alone should not be presumed to restore the mechanical behavior of an intact abdominal wall.

Panniculectomy may improve access or remove compromised tissue, but it adds a wound burden. A 2024 meta-analysis associated concomitant panniculectomy with increased wound morbidity and reoperation in ventral hernia repair. Those data support selective use and careful counseling, not a blanket prohibition or an EAF-specific effect estimate [36].

Loss of domain and elective adjuncts

Loss of domain describes an unfavorable relationship between herniated contents and abdominal capacity, but published volumetric definitions are not interchangeable. Imaging should therefore specify the method used rather than report an unexplained percentage [37]. When reduction risks high pressure or respiratory compromise, preparation and intraoperative reassessment become particularly important.

Botulinum toxin A and progressive preoperative pneumoperitoneum can assist selected elective loss-of-domain reconstructions. A 2024 systematic review of combined use reported feasibility, but predominantly nonrandomized evidence, variable protocols, and pneumoperitoneum-related complications restrict confidence in comparative benefit. These adjuncts are not established acute-OA or active-EAF treatments and should not be presented as a standard addition to temporary closure [38].

Outcomes, surveillance, and evidence gaps

Fistula healing must be distinguished from spontaneous closure and from restoration of intestinal autonomy. In a 2022 ECF meta-analysis of 53 studies and 3,078 patients, reported healing was 89%, but 72.5% of patients in studies reporting treatment underwent surgery. Recurrence after initially successful treatment was 11.1%, and mortality was 8.5%. These are heterogeneous treatment-pathway outcomes, not estimates of spontaneous EAF closure [39].

Reported recurrence varies by treatment pathway and population. The surgical timing review reported pooled postoperative recurrence of 19% (95% CI 15%-24%), the contemporary specialist ECF cohort reported 24%, and the 2026 combined-reconstruction synthesis reported fistula recurrence of 9% (95% CI 5%-16%). Different populations, interventions, outcome denominators, and follow-up explain why these estimates should be displayed separately rather than merged or directly ranked [15,17,22].

Evaluation should report bowel continuity, stoma status, dependence on intravenous support, fistula recurrence, anastomotic leak, infection, mesh complications, hernia or bulge, and further surgery. Abdominal pain, activity, return to work, body image, and quality of life complement anatomical outcomes. Long-term contaminated reconstruction cohorts demonstrate why hernia recurrence and the need for another hernia operation are distinct endpoints; surveillance limited to 30 or 90 days misses later structural failure [20].

The main unresolved questions are the optimal selection and timing of combined reconstruction, comparative fistula-isolation performance, objective readiness measures, durability of resorbable reinforcement, and recovery of abdominal wall function. Prospective studies should stratify EAF separately from tract-bearing ECF, record actual contamination and closure anatomy, and distinguish recurrent fistula, radiographic hernia, symptomatic failure, and reoperation.

## Conclusions

OA care, fistula treatment, intestinal rehabilitation, and abdominal wall reconstruction form one clinical pathway. Early decisions should protect bowel and preserve useful fascia, with traction considered in appropriate patients and safe closure pursued when the original indication has resolved. Established EAF requires controlled infection and drainage, measured replacement of losses, and recovery sufficient for definitive surgery. Timing should reflect clinical readiness and safe anatomy rather than a fixed interval alone.

Definitive reconstruction should address intestinal continuity, fascial mechanics, and soft-tissue coverage as separate but coordinated objectives. Randomized evidence supports synthetic over biologic reinforcement in selected controlled contaminated repairs, while most evidence on combined fistula reconstruction, release techniques, and flaps remains observational. Prosthetic bridging spans an unclosed fascial defect and may be necessary when continuity cannot safely be restored. Mesh exposed through a soft-tissue wound is a coverage complication requiring assessment of infection, bowel involvement, and salvage feasibility; deliberately leaving mesh uncovered is not a reconstructive goal. Durable success encompasses intestinal autonomy where achievable, a stable abdominal wall, acceptable function, and patient-defined recovery.
